# Numerical investigation of sand erosion rate in a horizontal axis wind turbine

**DOI:** 10.1016/j.heliyon.2024.e27676

**Published:** 2024-03-15

**Authors:** A.E. Abu El-Maaty, H.K. Abdallah, M.A. Kotb, R. Ben-Mansour, E.S. Alatawi

**Affiliations:** a Mechanical Engineering Department, KFUPM, Dhahran, 31261, Saudi Arabia; b Interdisciplinary Center for Renewable Energy and Power Systems, KFUPM, Dhahran, 31261, Saudi Arabia; c Mechanical Engineering Department, Tabuk University, Tabuk, 71491, Saudi Arabia

**Keywords:** Wind turbine, Horizontal axis, Small scale, Renewable energy, Wind energy, Sand, Erosion

## Abstract

Renewable energy represents an important alternative solution for many energy problems nowadays and a tool for a healthier environment by reducing carbon footprints resulting from burning fossil fuels. However, more work needs to be done towards maximizing the energy produced from renewable energy methods and making sure that the infrastructure used stays in service for a longer duration. Sand erosion phenomena is responsible for the degradation of the wind turbine blades and hence the decrease in their performance and life. In the current research, a numerical study of both performance and sand erosion of a Small-Scale Horizontal Axis Wind Turbine (SS-HAWT) is carried out. This study introduces new sights of instantaneous and forecasted erosion rates within the blade of the wind turbines. Three-dimensional E216 airfoil blades of radius 0.5 m are established according to blade element momentum theory. Sand particles with different mass flow rates of 0.001, 0.002 and 0.003 kg/s and uniform diameters of 50, 100 and 200 μm have been selected as eroding particles under two different average air velocities of 8 m/s and 10 m/s. The results indicate that the performance of wind turbines is enhanced as the flow separation at the suction side is shifted to the trailing edge. Furthermore, the optimum tip speed ratio is about 5 at an air velocity of 8 m/s with a power coefficient of 0.432. In terms of erosion findings, V-shaped scars are reported near the leading edge of the blades. In addition, the instantaneous erosion rate grows exponentially with the tip speed ratio. Therefore, the yearly prediction of maximum erosion depth at the optimum operating conditions is obtained to be 5.7 mm/year in some spots of the turbine blades.

## Nomenclatures and abbreviations

C_p_: Power coefficient
C_t_: Thrust coefficient
V: Airstream speed vector, m/s
ρ: Density, kg/m^3^
φ: Property needed for the investigation
${\vec{V}}_{g}$: Mesh velocity of the motion mesh, m/s
Γ: The diffusion parameter divided by the specific heat at a constant volume
$S_{\varphi }$: Source term
μ: Dynamic viscosity, Pa.s
λ: Tip speed ratio,-
ω: Angular velocity, rad/s
R: The radius of the blade, m or correlation coefficient
$A_{face}$: The area of the cell near the wall, m^2^
$C\left(d_{p}\right)$: Diameter correlation
f(∝): Impact angle
b(ν): Relative particle velocity correlation
y^+^: Wall distance estimation

**Abbreviations**
BEM: Blade Element Momentum
SS-HAWT: Small-Scale Horizontal Axis Wind Turbine
CFD: computational fluid dynamics
ER: Erosion rate, mm/y
exp: Experimental
DPM: Discrete phase model
PSD: Particle sand distribution
RANS: Reynolds-Averaged Navier-Stokes

## 1 Introduction

Nowadays, the world is searching for alternative resources of energy. Wind energy as a renewable energy source has taken a lot of consideration in recent decades [1]. Wind turbines represent a promising tool to harness wind energy as an alternative energy source to fossil fuels [2].

HAWT takes the post of providing the most available wind energy. Furthermore, small-scale turbines have gained more popularity over the last decade [3]. Wind turbine blade design is the most essential design factor for judging the performance of wind turbines. The blade design is often designed based on the Blade Element Momentum Theory (BEM). This theory is utilized for estimating the chord and twist distribution along the spanwise of the blade which represents the rotor blade design. Many researchers carry out studies to modify the blade's performance as a way to enhance its performance. Kaya et al. [4] investigated experimentally and theoretically backward and forward-swept blades. The operating velocity was 11 m/s with a rotor diameter of 0.9 m. Regarding the power coefficient (C_p_), the forward type is found to be more effective, while the backward type experienced a low thrust coefficient (C_t_). MacPhee et al. [5] studied the impact of using flexible and rigid blade turbines to enhance the performance of a SS-HAWT. They used three blades of 1.142 m diameters and an airfoil of type (NACA 0015). They found that flexible blades improve performance more than rigid ones. Ibrahim et al. [6] suggested blades with slots starting at the leading edge and their main focus was to examine the power coefficients theoretically and experimentally in the presence of these slots. They found that slots increase the turbine performance as the wind speed is reduced. Abdelwaly et al. [7] studied the performance of shrouded and un-shrouded wind turbines with a blade airfoil type of S809 and a rotor diameter of 10.058 m. They applied Reynolds-Averaged Navier-Stokes (RANS) equations and an SST k-ω turbulence model. The results indicated that at a low wind speed turbine of nearly 5 m/s, an increase of 160% in the torque coefficient is achieved with shrouded blades. Many tests have been carried out to extend the range of the operating wind speed and to enhance the power outputs [8,9] by linearizing both the twist and chord of the airfoil. It was observed that the power coefficient increased by 3.33% with this technique. Moreover, Applying different airfoils in the blade geometry also achieved an enhancement in horizontal axis wind turbines [10,11].

Blade erosion is one of the major problems that occur in wind turbines over time and affects badly their performance. The issue contains progressive damage to the wind blades due to climatic and meteorological reasons. For instance, many wind turbines are located in various operating environmental conditions, such as offshore locations, hilly terrain and snow. All these locations have extreme sand contained in the air [12]. Harsh climates and high blade tip speed increase the amount of erosion susceptibility. Erosion may wear out the device's wall, which in turn results in high maintenance costs and loss in material weight. As many wind turbines are sited near uninhabited areas, sand erosion may represent a potential risk of wind turbine erosion.

However, sand wear is a sophisticated phenomenon that relies on different factors. Over the last decade, many erosion predictive theories have been suggested. About 28 erosion correlations identified 33 influencing variables with 5 parameters, on average, for each correlation [13]. Sand erosion depends strongly on carrier fluid and particle characteristics. In a gas-solid stream, sand particles penetrate the bulk fluid with a change in the flow direction. When the carrier fluid is liquid, the sand particles will follow the same direction as the fluid [14]. Additionally, the erosion rates and patterns are strongly dependent on the type of fluid. A V-shaped pattern is expected in the gas-solid flow while it is not fixed in liquid [15]. Regarding wind turbines, Rempel [16] concluded that the wind turbine starts to show signs of erosion after an operating period of three years. The potential damage exists after five years without the required maintenance. To investigate the impact of surface roughness, Ehrmann et al. [17] performed a wide range of experiments on different blades' roughness and repeated these experiments on an airfoil in a wind tunnel. Gaudern [18] performed various experimental tests on an 18% thick commercial wind turbine airfoil. The study involved measuring both drag and lift coefficients at different erosion levels. Maniaci et al. [19] established a computational model to observe the influence of roughness and erosion over the wind turbine blades. Shankar Verma et al. [20] found that offshore rainfall conditions cause a higher rate of erosion damage for wind turbine blades than onshore conditions. Wang et al. [21] performed a computational fluid dynamics (CFD) analysis of an eroded wind turbine and found that the erosion level significantly affects wind turbine power coefficient (Cp) and flow separation. Maximum lift force is reduced by 25% as a result of erosion in the leading edge of the wind turbine while the minimum drag force increased by 60%. It is also observed that stall angle has appeared with a lower angle of attack [22]. Sareen et al. [23] studied experimentally the influence of erosion on airfoils. The experiments were conducted at two different Reynolds numbers of 1.85 × 10^6^ and 1 × 10^6^. They found that the lift coefficient decreased by 17%, while the drag coefficient increased by 400% with heavy erosion. Some attempts are performed to protect the leading edge from erosion by special materials. Weigel [24] tested a protection design to protect helicopter blades from rain and sand wear. He could reduce the erosion impact using this system. Still, wind turbine blades are different from helicopters because of different flow behaviour and rotor speeds.

Based on previous studies, The alterations of the leading-edge design of the wind turbine due to harsh conditions are gaining growing interest because of the significant effect on operation, maintenance, energy cost, and aerodynamic performance degradation [1]. In terms of renewable energy sustainability, wind turbine erosion reduces the potential of using wind turbines as sustainable energy due to periodic maintenance and low performance. The leading edge erosion phenomenon is a subtractive case and usually occurs due to the high-speed collision of the wind turbine blades with other solid or liquid particles like hail stones and water mist [10]. For more clarification, the peripheral tip speed of the wind turbine blades reaches about 90–100 m/s, with higher velocity being appealing due to many causes, involving higher aerodynamic performance, and lower cost of the gearbox [11]. The key interest in this study is the erosion of wind turbine blades which is still an unresolved issue in the industry. The main challenge with an eroded wind turbine is low performance. Leading edge erosion raises the drag force and reduces the lift force acting on the airfoil, consequently decreasing the torque output from the rotor. As a result, lower power output and subsequently the annual energy from the wind turbine is reduced. Moreover, this increases the operating and maintenance costs for the wind turbine [13]. Investigating the erosion issues will push up the wind turbine performance and will contribute towards more wind energy sustainability.

To the best of the author's knowledge, erosion rates using CFD modelling of non-eroded small-scale wind turbines have not been investigated yet. Moreover, CFD is considered a powerful tool to simulate the performance of the wind turbine and predict the flow behaviour along the airfoil blades.

Therefore, the novelty of this work is to examine the performance of non-eroded wind turbines (E216 type) at different tip speed ratios in addition to giving an estimation of potential mass losses due to erosion using numerical analysis. Computational results of both performance and erosion rate will be also validated with other experimental and CFD results. This work introduces the instantaneous and predicted erosion rates that occurred in the wind turbine blades. This could be useful for estimating the period of maintenance and keeping the performance of the wind turbine relatively high.

The skeleton of the paper starts with the proposed geometry section that involves the exact details of the wind turbine and the domain characteristics, then the mesh generation section highlights the details of mesh generated, Y+ values on both sides of the wind turbine and the mesh sensitivity results. After that, the numerical methodology contains the details of the CFD model and discretization scheme. Furthermore, the model validation section is added to validate the current CFD model with previously published experimental results. Moreover, the erosion model setup is introduced to highlight the steps and the model used to obtain the erosion rate values. Finally, erosion model validation is performed to show the reliability of the current erosion rate model compared to previous CFD and experimental erosion rates, and the obtained results are discussed.

## 2 Proposed geometry

**Fig. 1:**
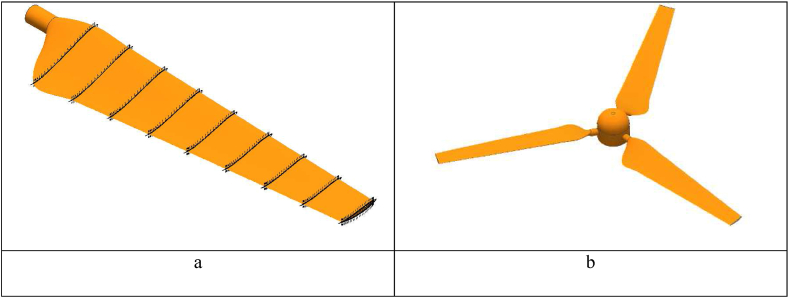
a- View of the blade b- The wind turbine.

**Fig. 2:**
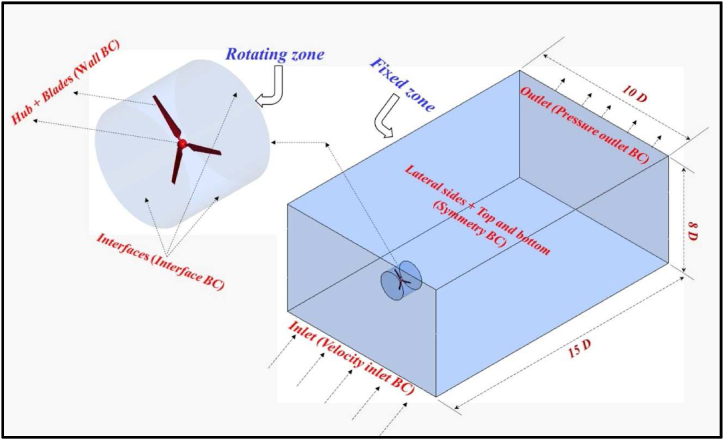
Wind turbine system with the stator part specification.

In this section, the proposed geometry of the rotor blades is obtained from Ali et al. [25]. The chord and twist distribution is linear. The chord starts from 0.09 m at the hub and ends with 0.045 m at the tip of the blade. The twist angle starts at 16.7° at the hub region and ends at 4.2° at the tip. Every airfoil plane is scaled and twisted depending on the distance from the hub. The airfoil of E216 is proposed for the blade design which has a length of 0.5 m as shown in Fig. 1a. INVENTOR 2016 software is used for the drawing of the blades and the hub as shown in Fig. 1b. The stator and the rotor are shown in Fig. 2 [26,27]. According to the dimensions of the rotating zone, it is worth mentioning that, the height of the cylinder is 1D and the diameter is 1.2D [26] and the operating conditions of the HAWT are indicated in Table 1.

**Table 1:** Data for different operating parameters.

Wind speed	8–10 m/s
Tip speed ratio	4
Airfoil type	E216
Max lift-to-drag ratio	68.534
Blade radius	0.5 m
Reynolds number	100000–150000
Attack angle	5°
Number of blades	3 blades

### 2.1 Mesh generation

**Fig. 3:**
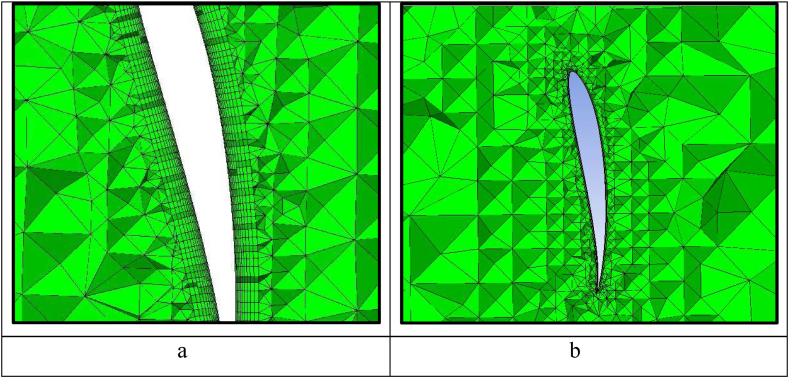
Mesh density around A-the wind turbine and B-the airfoil.

**Fig. 4:**
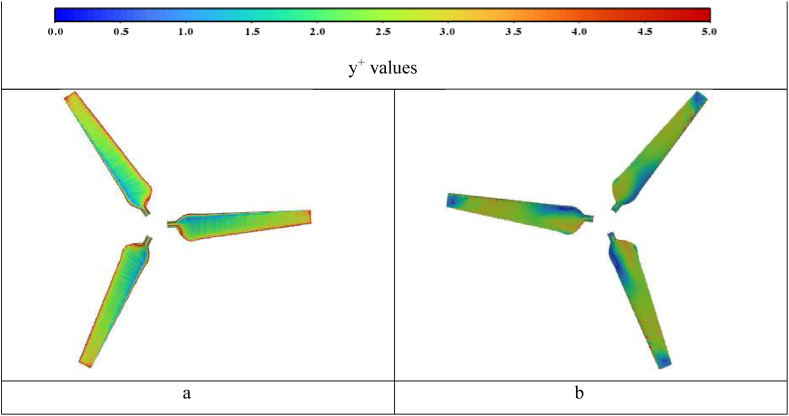
y^+^ values for a-front view b-back view.

In this study, the sliding mesh motion method [28] is selected to simulate the wind flow throughout the whole system. Mesh is intensified near the airfoil blades and the hub to capture the flow behaviour near the blade wall such as shear stress and flow separation. Table 2 indicates the input parameters to set up the mesh using ANSYS ICEM program, while Table 3 indicates the quality of the mesh exported to the fluent. It is observed that the number of cells is about 3 million cells, therefore a workstation is used for this simulation. The aspect ratio of the mesh is about 20 for the overwhelming majority of the cells, nearly 99%. Fig. 3a and b highlight the final shape of the wind turbine and airfoil mesh. Mesh smoothing and density around the blades are also performed. Near the blade surfaces, 10 inflation layers are created to secure an acceptable value of dimensionless wall distance y^+^. The resulting y+ values were near 5, as indicated in Fig. 4a and b for front and back surfaces.

**Table 2:** Mesh setup parameters.

Maximum element size	0.04
Maximum blade element size	0.002
Max hub size	0.002
No. of inflation Layers	10
Initial height	0.000123
Height ratio	1.2

**Table 3:** Mesh quality.

Total number of the elements	3096350
Minimum volume (m3):	3.515640e-13
Maximum volume (m3):	6.801973e-03
Aspect ratio indices	
Max 20.6355	378830 (12.235%)
Max 10.31775	2684474 (86.698%)

## 3 Mesh sensitivity study

**Fig. 5:**
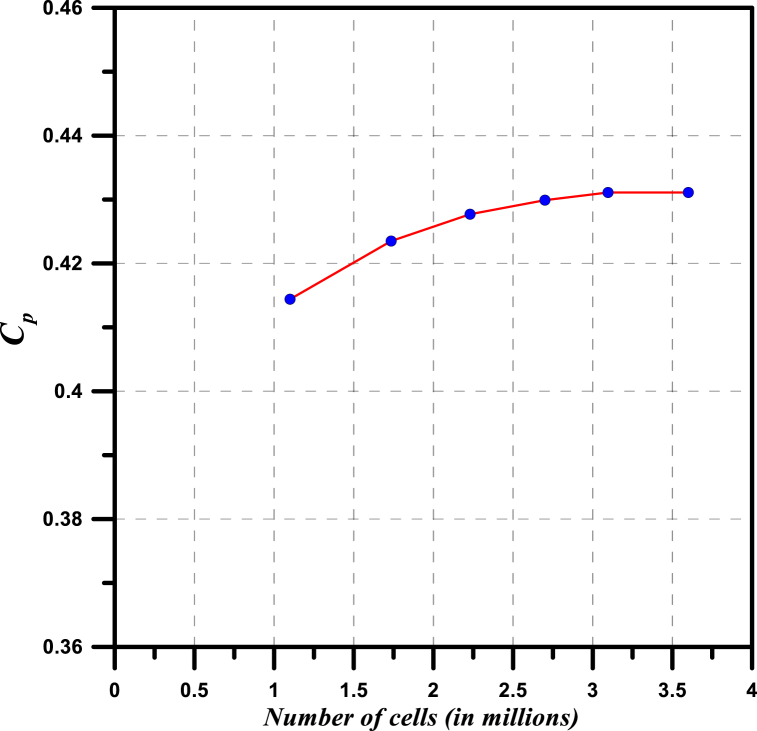
Effect of grid resolution on the power coefficient at a tip speed ratio of 5, V = 10 m/s.

A grid independence study is performed for the proposed wind turbine using Ansys ICEM program with different mesh sizes, to ensure that raising the grid size does not affect the obtained results. Therefore, mesh size increases from 1.3 million cells up to 4.2 million cells, as shown in Fig. 5. It is also noticed from Fig. 5 that, after the number of cells of 3.096 million cells, the performance remains almost constant, hence a grid of 3.096 million cells is utilized for wind turbine simulations.

## 4 Numerical methodology

The numerical procedure is presented in this study to estimate the flow characteristics around the SS-HAWT. The applied governing equations are discretized by the finite volume scheme using Ansys Fluent 2022 [28]. Solution methods are selected as shown in Table 4 while Table 5 indicates the discretization scheme. The SST k-ω turbulence model is employed to satisfy the RANS equations. It has been proven that this model has a good prediction of simulation behaviour around the HAWT [[29], [30], [31]]. The employed numerical procedure assumed that the flow is Newtonian, incompressible, 3D unsteady turbulent with constant fluid properties. The conservation equations applied are using Eqs. (1), (2), (3), (4).

(1) $$\nabla .\left(\vec{V}-{\vec{V}}_{g}\right)=0$$

(2) $$\rho \frac{d}{dt}\int \varphi dV+\rho \int \varphi \left(\vec{V}-{\vec{V}}_{g}\right).d\vec{A}=\int \Gamma \nabla \varphi .d\vec{A}+\int S_{\varphi }dV$$

Where:

**Table 4:** Solution parameters.

General	Solver (Pressure-based) unsteady
Time step	0.001 s
Max Iteration/Time step	20
Model	Viscous-SST k-ω
Materials	Air
Cell zone conditions	Fluid (air) mesh motion Reference frame (speed N 193 rpm or w = 20 rad/s) The fluid stator is air
Pressure-velocity coupling	SIMPLE (semi-implicit Method for Pressure-Linked Equation)

**Table 5:** Discretization schemes.

Solution methods	SIMPLE
Spatial discretization (Gradient)	Least square cell-based
Pressure	Second order
Momentum	Second order upwind
Turbulent Kinetic energy	First order upwind
Specific dissipation rate	First order upwind
Transient formulation	First order implicit

φ is the property needed for the investigation

$\vec{V}$ is the air stream speed vector

${\vec{V}}_{g}$ is the mesh velocity of the motion mesh

Γ is the diffusion parameter divided by the specific heat at a constant volume

$S_{\varphi }$ is the source term

ρ is the fluid density

Turbulence kinetic energy k equation

(3) $$\rho \frac{d}{dt}\left(k\right)+\rho \nabla .\;\left[k\left(\vec{V}-{\vec{V}}_{g}\right)\right]=\nabla .\;\left[\left(\mu +\mu_{t}/\sigma_{k}\right)\nabla k\right]+G_{k}-Y_{k}$$

Specific dissipation rate ω equation

(4) $$\rho \frac{d}{dt}\left(\omega \right)+\rho \nabla .\;\left[\omega \left(\vec{V}-{\vec{V}}_{g}\right)\right]=\nabla .\;\left[\left(\mu +\mu_{t}/\sigma_{\omega }\right)\nabla k\right]+G_{\omega }-Y_{\omega }$$

Tip speed ratio is the ratio between peripheral speed at the tip and free stream wind speed, We could manage the value of tip speed ratio by controlling the angular velocity of the blade and under different air velocities. The following Eq. (5) describes the tip-speed ratio correlation

(5) $$\lambda =\frac{wR}{V}$$

λ is the tip speed ratio

ω is Angular velocity, rad/s

R is The radius of the blade, m

V is Air velocity, m/s

## 5 Model validation

**Fig. 6:**
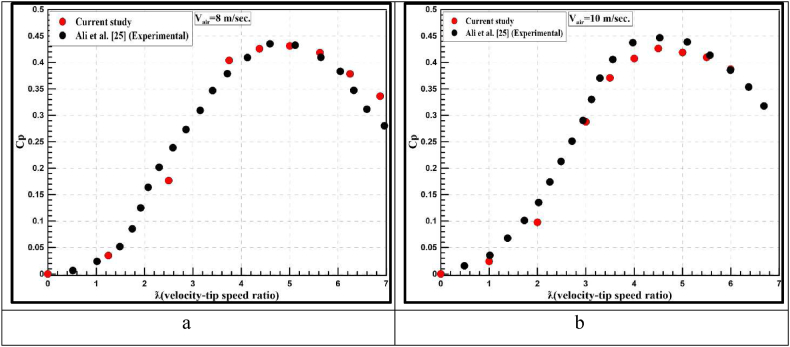
Model validation at A- 8 m/s wind speed and B- 10 m/s wind speed.

Fig. 6a and b shows the numerical results of the power coefficient from this study compared with the experimental results obtained by Ali et al. [25,26]. For all values of tip speed ratios, the current results showed good agreement with the experimental work [25].

To get the overall deviation between the current and experimental results, the correlation coefficient technique is applied in the present study [27,32]. The R^2^ coefficient is estimated using Eqs. (6), (7)) as follows [33]:

(6) $$R^{2}=1-\frac{\sum {\left(\varphi_{\exp}-\varphi_{\;current\;study}\right)}^{2}}{\sum {\left(\varphi_{\;\exp}-{\bar{\varphi }}_{\;\exp}\right)}^{2}}$$

(7) $${\bar{\varphi }}_{\;\exp}=\frac{\sum_{i=1}^{n}\varphi_{\;\exp}}{n}\text{where}\;n\;\text{is}\;\text{the}\;\text{number}\;\text{of}\;\text{data}$$

Table 6 indicates the relative error values of the power coefficient and correlation coefficient at different values of λ. It is observed that the values of relative errors experienced low values, which assures the reliability of the current results. Additionally, the correlation coefficient values give confidence in the present simulation with a good resolution.

**Table 6:** Relative error values between the current study and [25].

v_air_ = 8 m/s					v_air_ = 10 m/s				
λ	C_p_ (current study)	C_p_ [25]	Relative error,%	R^2^	λ	C_p_ (current study)	C_p_ [25]	Relative error, %	R^2^
1.25	0.0350	0.0379	7.57	0.986	1	0.024074	0.030636	21.42044	0.962
2.5	0.1764	0.227	22.25		3	0.287766	0.290492	0.93868	
3.75	0.4036	0.3786	6.60		4	0.407272	0.437346	6.876416	
4.375	0.4258	0.4352	2.15		4.5	0.426206	0.446648	4.576822	
5	0.4311	0.4323	0.283		5	0.418822	0.438851	4.563856	
5.625	0.4186	0.4097	2.16		5.5	0.409642	0.413611	0.95978	
6.25	0.3783	0.3474	8.90		6	0.387009	0.385431	0.40933	

## 6 Erosion model setup

The main factor of erosion rate is the material particle impingement characteristics. The nodes next to the wall register the impact characteristics of individual sand particles. The rate of erosion could be calculated using Eq. (8) as follows:

(8) $$R_{erosion}=\sum_{p=1}^{No\;of\;particles}\frac{{\dot{m}}_{p}C\left(d_{p}\right)f\left(\propto \right)\nu^{b\left(\nu \right)}}{A_{face}}$$

Where $A_{face},C\left(d_{p}\right),f\left(\propto \right)and\;b\left(\nu \right)$ are the area of the cell near the wall, diameter correlation, impact angle, impact angle function and relative particle velocity correlation. f(∝) was calculated depending on whether the material is ductile or brittle. The blade material used in this study is stainless steel. To set up the erosion model in Fluent, first, it is better to run all non-eroded wind turbine cases and after reaching a steady state case, we should activate the erosion model using the discrete phase model (DPM). The maximum tracking parameter is 50000 by default with a 0.01 m length scale. In terms of physical models,

## 7 Erosion model validation

To the best of the author's knowledge, literature about erosion in wind turbines is limited by large scale while only a few references describe the erosion rate within large-scale wind turbines due to water droplets [34]. Since no experimental results are available to estimate erosion in wind turbines, authors have to validate one of the available erosion models such as Generic, Finnie, Mclaury, Oka, and DNV against another available experimental work. This approach is critical for determining the best model for air sand erosion. In this regard, Wee and Yap [35] carried out CFD study to estimate the air-sand erosion rate in the elbow. In their study, they validated their erosion model with another experimental study [36]. Therefore, it is proposed to validate the erosion numerical model with these two studies [35,36].

**Fig. 7:**
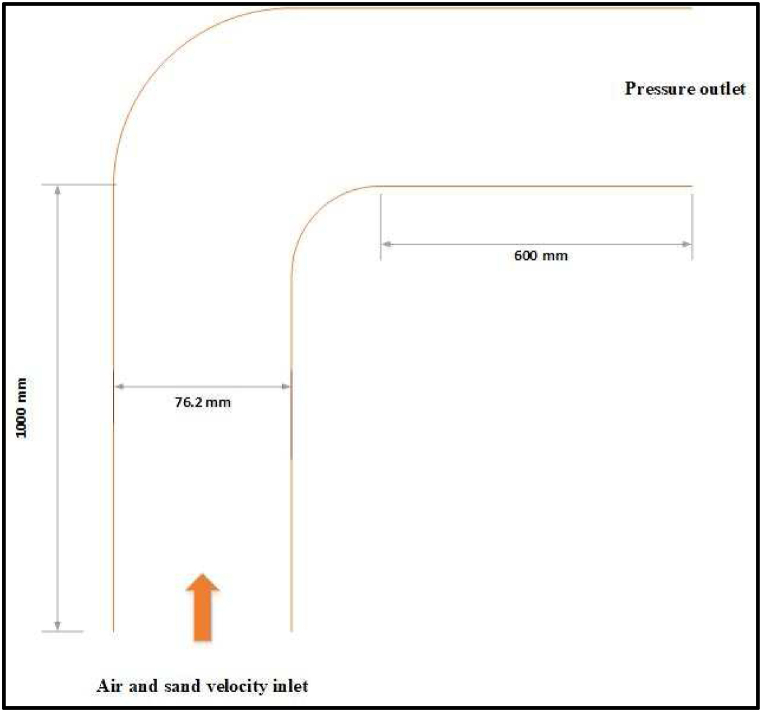
Schematic diagram of the elbow.

Fig. 7 shows a 90° elbow which is used in the validation process. The vertical length of the elbow is 1.0 m with a 0.0381 mm internal radius connected to a 0.1143 mm radius curvature with 0.6 m horizontal length. Stainless steel 316 is selected as the wall material. The sand and air properties used in this validation and their characteristics are listed in Table 7, Table 8. Previous investigations [37] assured that there is a necessity to involve particle size distribution during the forecasting of wear rate. Therefore, particle size distribution and uniform size models in discrete phases are investigated in this study. The injection properties and operating conditions are set as shown in Table 9. The inlet velocity is selected as normal to the boundary and the outlet is zero gauge pressure with turbulence intensity of 5 % and hydraulic diameter equivalent to the elbow diameter. The air and sand flow conditions applied for this validation are listed in Table 10.

**Table 7:** Sand particles properties.

Size distribution model	Sand properties	Values
**Set 1** Particles size distribution model [30] (Rosin Rammler Equation)	Minimum diameter	65 μm
	Maximum diameter	300 μm
	Shape factor	0.53
	Spread diameter	4.1
	Mean diameter (PSD)	177 μm
**Set 2** uniform size distribution [30]	Uniform diameter	150 μm

**Table 8:** Fluid properties.

Carrier fluid	Density	Viscosity
Air	1.125 kg/m^3^	1.8*10^−5^ kg/m.s

**Table 9:** Injection properties of sand erosion in 90° elbow.

Injection type	From the surface (inlet)
Inert particles	Sand
Density	1700 kg/m^3^
Inlet velocity (sand)	23 m/s
Diameter Distribution	Uniform (set 1) Rosin-rammler (set 2)
Diameter	Uniform (150 μm) (set 1) Particle size distribution (PSD 177) (set 2)
Turbulent dispersion	-Activate discrete random walk model -Activate Random eddy lifetime

**Table 10:** Air and sand flow rates.

Set 1			Set 2		
Air velocity	Particle size	Sand flow rate	Air velocity	Particle size	Sand flow rate
11	PSD 177	0.00294	11	150 μm	0.00294
15	PSD 177	0.00274	15	150 μm	0.00274
23	PSD 177	0.00297	23	150 μm	0.00297
27	PSD 177	0.00238	27	150 μm	0.00238

## 8 Validation results

Outputs are validated by estimating the relative error percentage between the current model results and two previous studies: the experimental study [36] and the numerical study [35]. The relative error is calculated based on the maximum erosion rate obtained by each model. The experimental results [36] are obtained from the ultrasonic transducer reading based on the same four air velocities mentioned in Table 10 using the same diameter of 150 μm. We could convert the erosion rate from the steady erosion model from kg/m^2^.s to mm/year using the following Eq. (9).

(9) $$ER\;\left(mm/year\right)=\frac{24*365*3600*1000*ER\;\left(kg/m^{2}.s\right)}{desnity\;of\;steel\;\left(7990\;kg/m^{3}\right)}$$

**Fig. 8:**
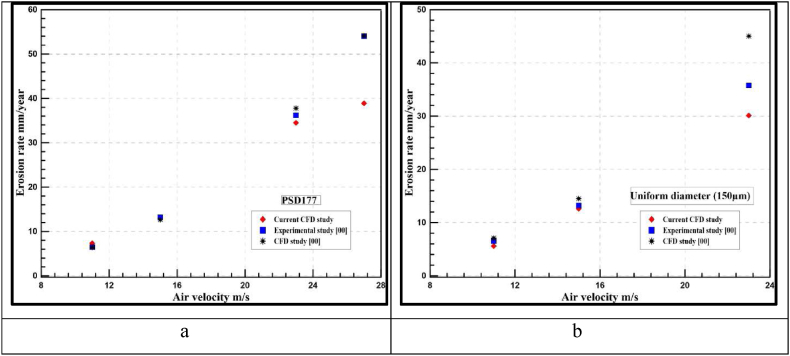
Erosion model validation for a- PSD 177 b-uniform diameter of 150 μm.

**Fig. 9:**
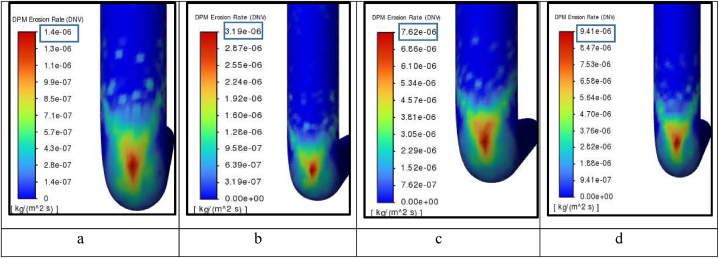
Erosion rate contours for uniform diameters and air velocity of a) 11, b) 15, c) 23, d) 27 m/s.

As shown in Fig. 8a and b, erosion rates of the current study showed a good agreement with the two studies up to air velocities of 23 m/s for uniform-size particles and PSD 177. Depending on the findings and comparison between different erosion models, DNV erosion model results have been proven to have the highest accuracy among other erosion models in the case of air-sand erosion up to 20–23 m/s air velocities. Fig. 9a, b, 9c, and 9d indicate the values of the maximum instantaneous erosion rate for different air velocities. From the graph, we can observe that the impact between the wall and the sand particles is V-shaped and this was previously published [36] which also assures the reliability of the DNV results.

## 9 Results and discussion

**Fig. 10:**
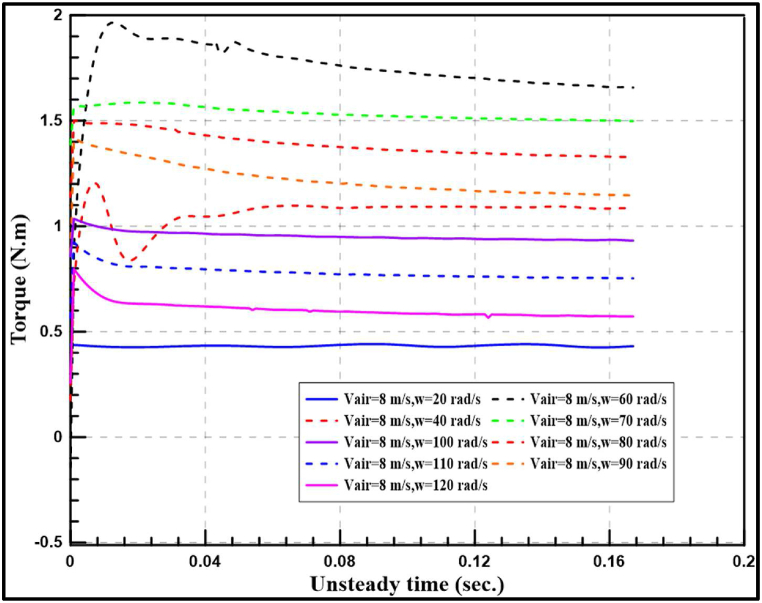
Transient and steady produced torque at different angular tip velocities and 8 m/s wind speed.

**Fig. 11:**
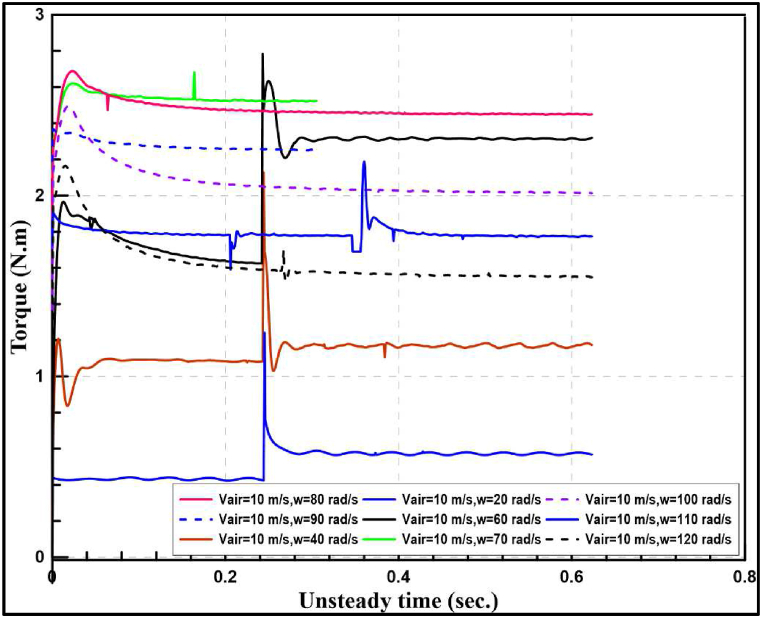
Transient and steady produced torque at different angular tip velocities and 10 m/s wind speed.

Fig. 10, Fig. 11 indicate the time required for reaching steady torque at different tip speed ratios for wind speeds of 8 and 10 m/s. It is noticed that the maximum torque is generated at approximately angular tip velocity of 60 rad/s for wind speed of 8 m/s and between 70 and 80 rad/s for 10 m/s. While at 20 rad/s angular velocity produced the minimum torque for the two wind speeds.

**Fig. 12:**
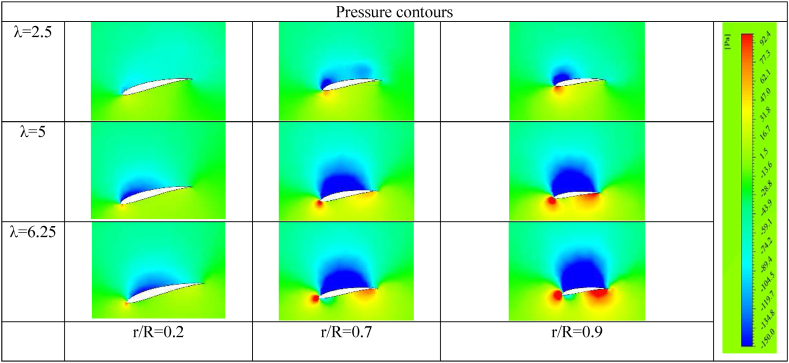
Pressure contours around the airfoil sides at air velocity 8 m/s.

**Fig. 13:**
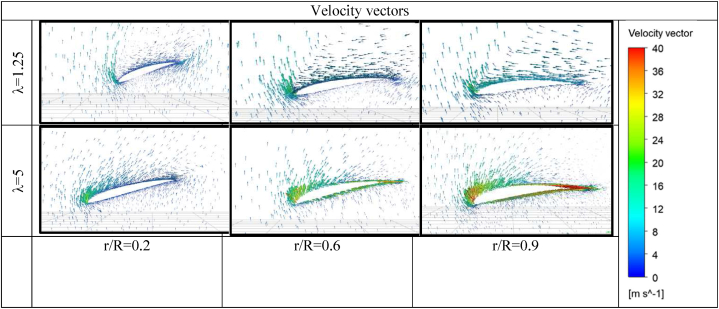
Velocity vectors around the airfoil sides at air velocity 8 m/s.

Simulation of air movement on both sides of the airfoil represents an important factor to observe the air behaviour and relate that to the performance. Static pressure contours and velocity vectors are investigated at different cross-sections of the airfoil at an inlet air velocity of 8 m/s as shown in Fig. 12, Fig. 13. Fig. 12 indicates the pressure contours around the airfoil for these conditions; air velocity of 8 m/s, tip speed ratios of 2.5, 5, 6.25 at 20%, 60% and 90% distance from the hub. From the graph, it is clear that pressure increases on the front side since the fluid comes to near-stagnation. The opposite occurs on the suction side, as the flow speed increases, the pressure decreases as well. At λ = 2.5, the performance is poor due to the flow separation occurring as a result of the adverse pressure. At λ = 5, the flow succeeds to go over the back side without separation, therefore it is logical to achieve high performance.

Fig. 13 highlights the velocity vectors on the airfoils at different sections and tip speed ratios. At a low tip speed ratio λ = 1.25, it is evident that many vortices are formed and fill the majority of the backside surface as the flow separation occurs near the leading edge. As the blade goes faster, the vortices are damped on the suction side and the air becomes more tangent to the airfoil surface. It is more logical to expect low performance of the wind turbine at a low tip speed ratio due to early flow separation and the creation of many vortices at the suction side. As expected from the results, Table 11 indicates the exact values for blade angular velocity, generated torque, power, maximum power, and power coefficient.

**Table 11:** Turbine performance at air velocity 8 m/s and different radial velocities.

ω	torque	power	Max power	cp	Tip speed ratio
0	0	0	0	0	0
20	0.431361	8.62722	246.300656	0.035027	1.25
40	1.086638	43.4655	246.300656	0.176473	2.5
60	1.657055	99.42332	246.300656	0.403666	3.75
70	1.498368	104.8857	246.300656	0.425844	4.375
80	1.327461	106.1969	246.300656	0.431168	5
90	1.145708	103.1137	246.300656	0.41865	5.625
100	0.93187	93.18704	246.300656	0.378347	6.25
110	0.752929	82.82223	246.300656	0.336265	6.875
120	0.582347	69.88159	246.300656	0.283725	7.5

**Fig. 14:**
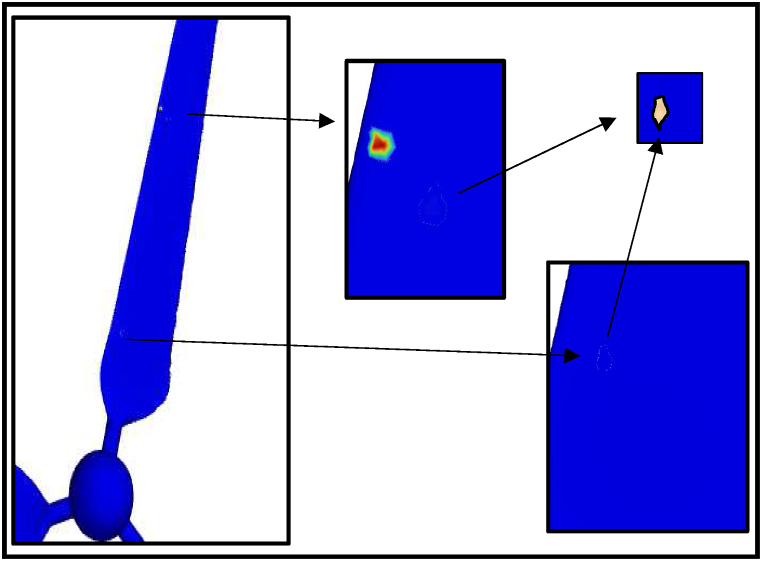
Erosion contours at 8 m/s of air velocity, 20 rad/s of angular velocity, sand flow rate 0.001 kg/s and particle diameters of 50 μm.

**Fig. 15:**
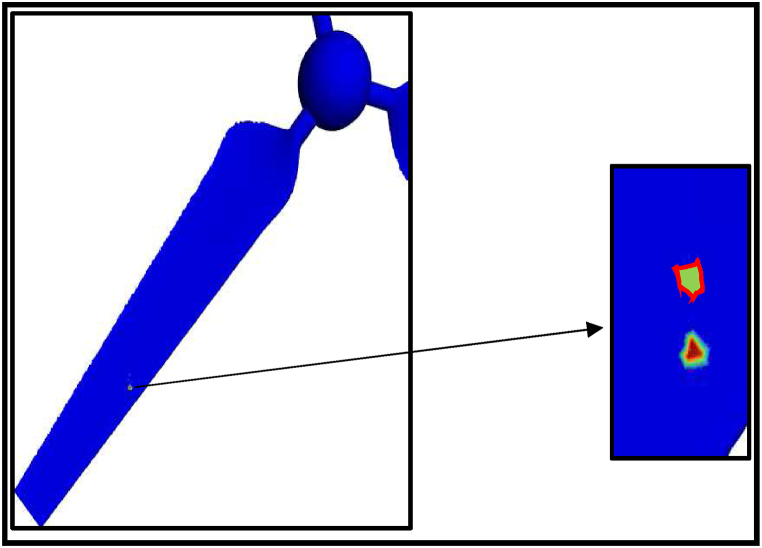
Erosion contours at 8 m/s of air velocity, 20 rad/s of angular velocity, sand flow rate 0.002 kg/s and particle diameters of 50 μm.

**Fig. 16:**
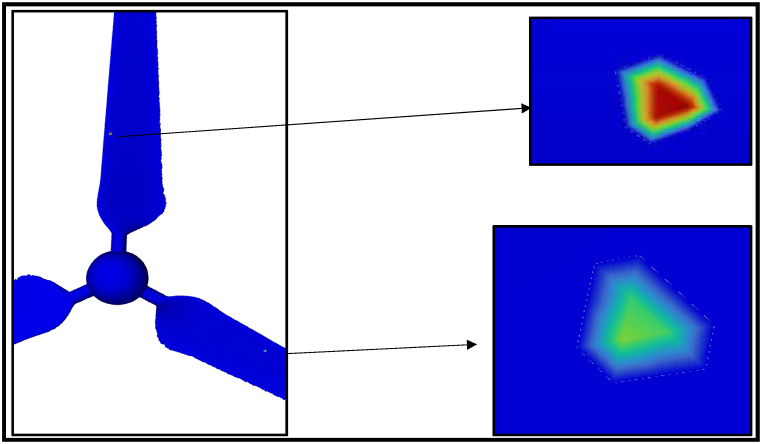
Erosion contours at 8 m/s of air velocity, 20 rad/s of angular velocity, sand flow rate 0.002 kg/s and particle diameters of 100 μm.

**Fig. 17:**
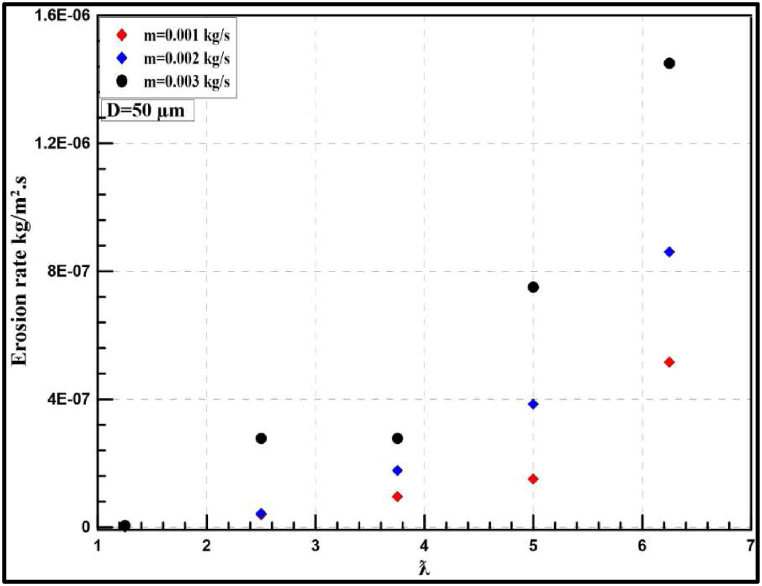
Instantaneous erosion rates for the wind turbine at 50 μm.

**Fig. 18:**
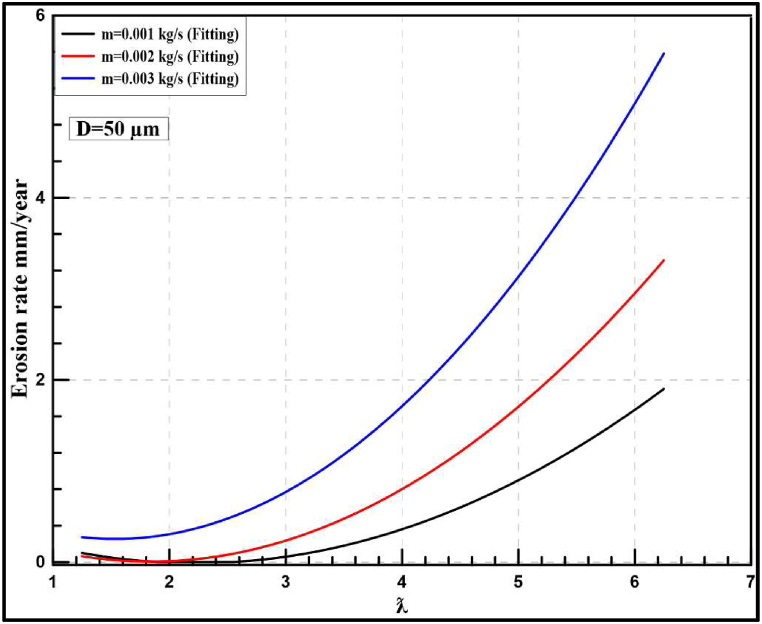
Yearly-prediction of erosion rates for the wind turbine at 50 μm.

The erosion rate in a gas-solid flow is investigated using CFD technique to indicate the instantaneous and yearly rates of sand erosion on a small-scale wind turbine. The following results are predicted from a sand impingement speed of 23 m/s. Stokes number is used to determine whether the sand will flow through the fluid streams or deviate from the fluid path. If the stroke number is near unity, the particle will follow the fluid streamlines [37]. Stokes number is reported for air sand flow from the history between 13000 and 33000. Based on the obtained results, the erosion rate of sand air flow is dependent on sand velocity, particle diameter and tip speed ratio. Fig. 14 shows the contour profile of the steady state erosion rate of sand air erosion. The profile is V-shaped as reported in the literature [14]. Different carriers may experience different erosion manners and patterns. In the case of liquid-solid flow, the erosion will depend on the relation between the fluid drag force and the particle inertia as well. For more clarification, the drag force will move the particle laterally before the impact with the wall resulting in comparatively less collision and erosion rate. In terms of erosion patterns and the case of liquid-solid flow, there is no fixed scar shape while V-shaped erosion is usually reported. The frequency of erosion locations appearing in Fig. 14, Fig. 15, Fig. 16 highlight that the leading edge is the most exposed location to erosion under different conditions. The erosion strength varies by location and it is maximum near the leading edge. To capture the erosion behaviour, Fig. 17 shows the rate of erosion at different tip-speed ratios and different mass flow rates at 50 μm. The instantaneous erosion rate grows exponentially with the tip speed ratio. In terms of impinged mass, the rate of erosion is more sensitive to impinged mass at a high-velocity tip speed ratio. According to optimum performance behaviour (λ = 5), the instantaneous erosion rate is 1.51E-07, 3.84E-07 and 7.51E-07 kg/m^2^.s for 0.001, 0.002 and 0.003 kg/s of injection particles, respectively. These values are estimated and fitted on an annual basis using exponential curve fitting to reach 0.59, 1.516, and 2.96 mm/year. This figure is doubled nearly with a particle diameter of 100 μm as shown in Fig. 18.

**Fig. 19:**
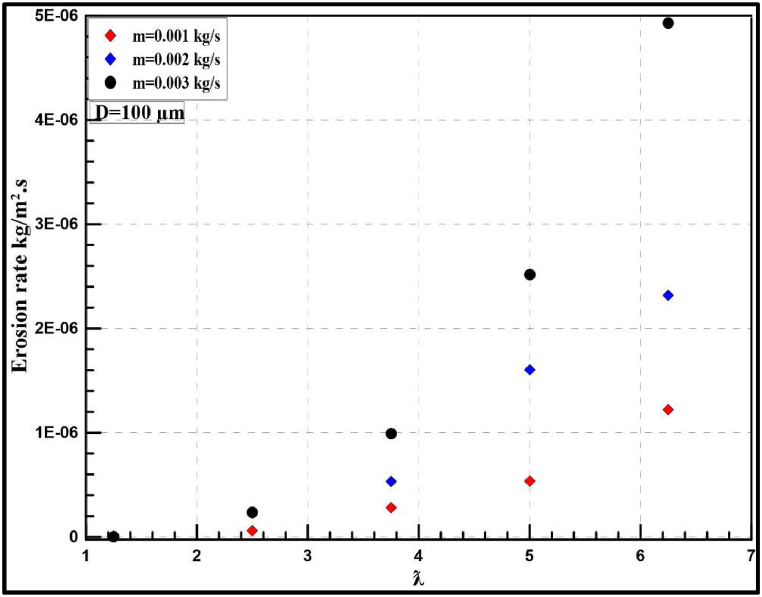
Instantaneous erosion rates for the wind turbine at 100 μm.

**Fig. 20:**
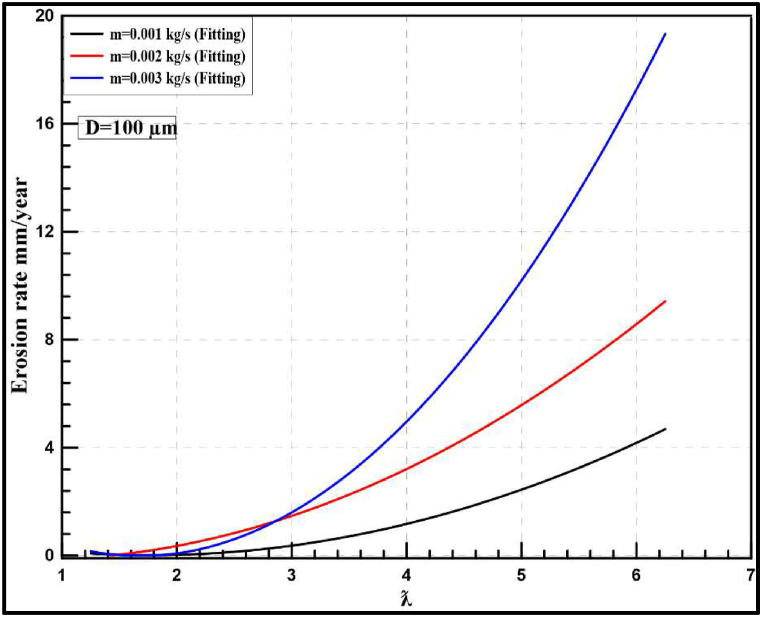
Yearly-prediction of erosion rates for the wind turbine at 100 μm.

The effect of increasing particle diameter for the same range of sand flow rates is also studied with various tip speed ratios. Fig. 19 depicts the instantaneous erosion rates for the wind turbine at 100 μm which show a similar trend to what is mentioned in the previous section but with a higher rate of erosion. According to optimum performance behaviour (λ = 5), the instantaneous erosion rate is 6E-07, 1.7E-06 and 2.6E-07 kg/m^2^.s for 0.001, 0.002 and 0.003 kg/s of injection particles, respectively. The annual rate of erosion is highly increased and the maximum erosion rate reached nearly 20 mm/year which is significantly higher than the previous results obtained at 50 μm. Additionally, the flow rate has a great effect on the erosion rate as shown in Fig. 20, increasing the flow rate from 0.001 kg/s to 0.003 kg/s results in an annual increase in erosion rate from 3 mm/year to 11 mm/year.

**Fig. 21:**
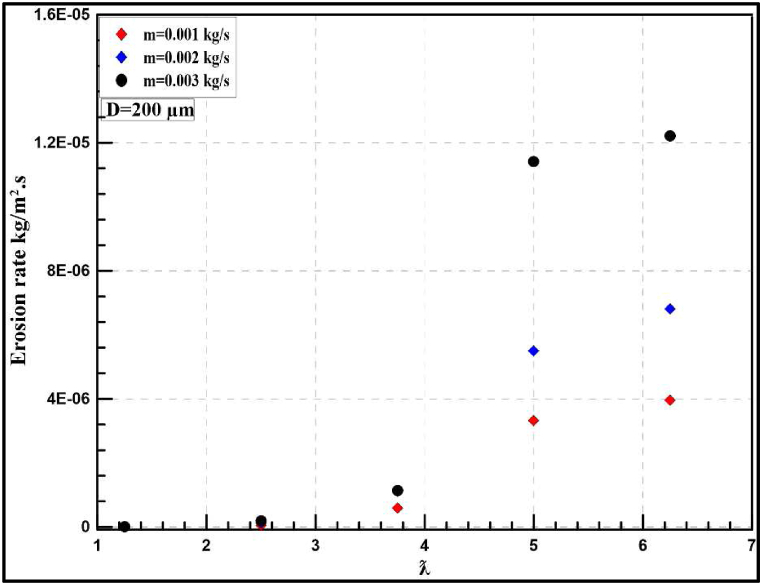
Instantaneous erosion rates for the wind turbine at 200 μm.

**Fig. 22:**
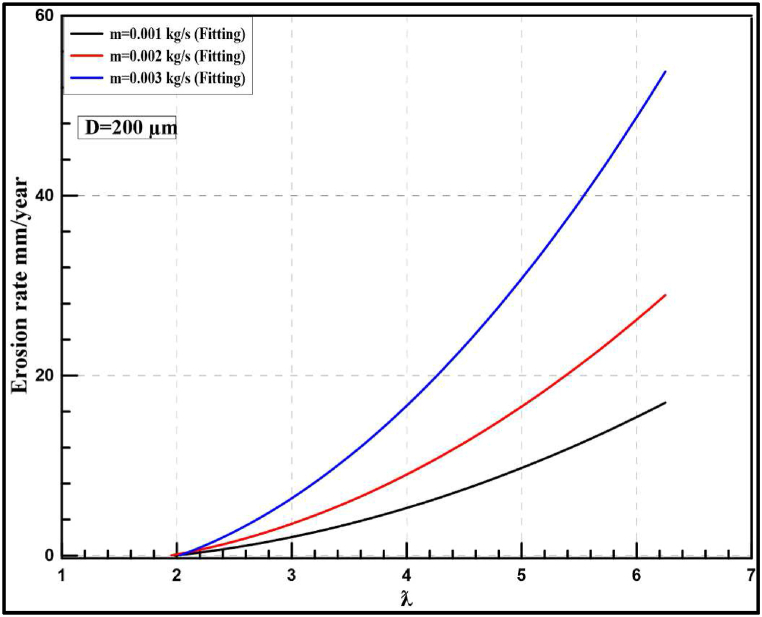
Yearly-prediction of erosion rates for the wind turbine at 200 μm.

**Fig. 23:**
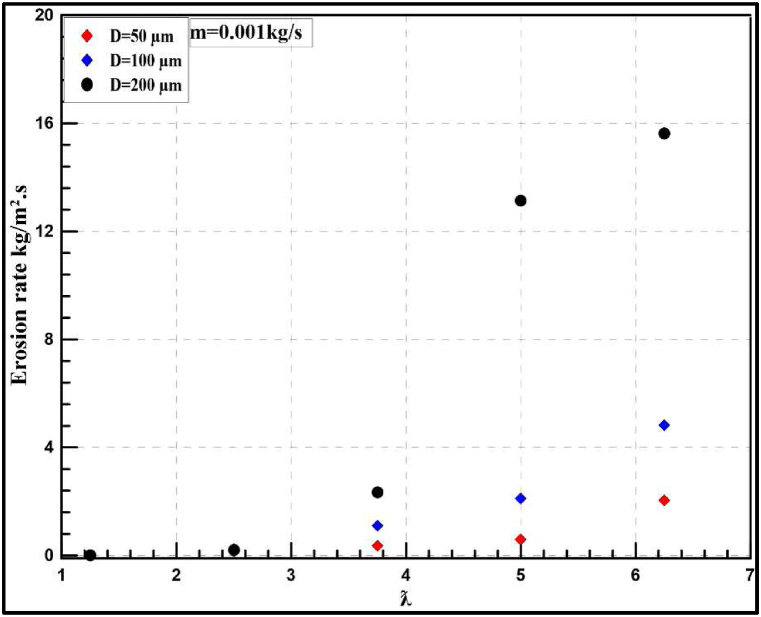
Instantaneous erosion rates for the wind turbine at 0.001 kg/s with different particle diameters.

**Fig. 24:**
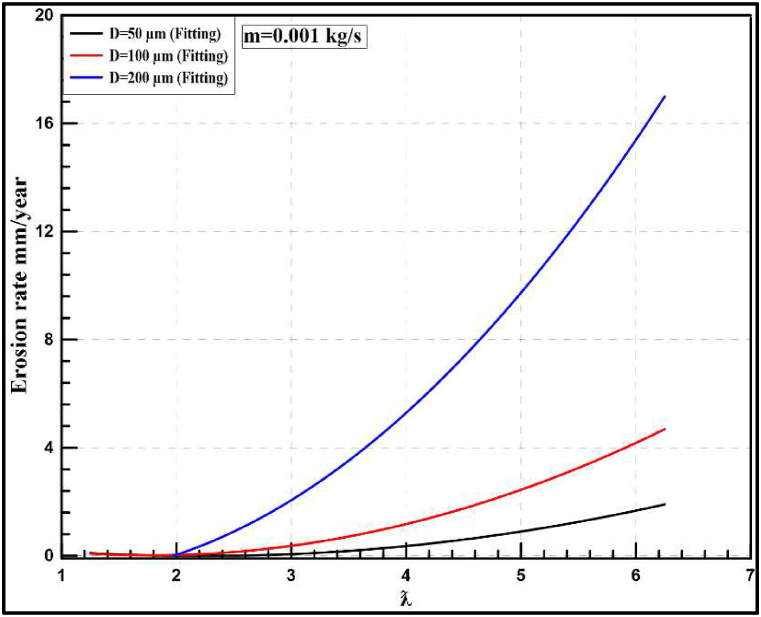
Annual erosion rates for the wind turbine at 0.001 kg/s with different particle diameters.

Fig. 21 shows instantaneous erosion rates at a particle diameter of 200 μm with different flow rates and tip speed ratios, at λ = 5. A sudden increase in erosion rate occurred for all the studied flow rates with a maximum value of 1.0E-05 kg/m^2^.s at 0.003 kg/s. Furthermore, Fig. 22 shows that the annual rate is increased sharply at particle diameter of 200 μm to reach 30 mm/year instead of 2.96 mm/year at 50 μm. Comparing the obtained results at λ = 5, authors notice that increasing particle diameter from 50 μm to 200 μm leads to an increase in the erosion rate by 12.5, 20.17 and 42.06 mm/year at mass flow rates of 0.001,0.002 and 0.003 kg/s, respectively. Finally, it is concluded that by increasing the diameter from 50 μm to 200 μm at the same flow rate, the erosion rate is highly increased. For more clarification, Fig. 23, Fig. 24 illustrate a comparison between the effect of studied particle diameters at the same flow rate.

## 10 Conclusion

In this study, the performance of SSHAWT as well as the sand erosion are numerically investigated. The radius of the wind turbine blade is 0.5 m. Additionally, the behaviour of the air in terms of pressure and velocity values and vectors around the airfoil is studied. The following conclusion can be made.

1 At a low tip speed ratio, the performance of the wind turbine is low due to early flow separation at the suction side.
2 This prototype could produce 106.4 W power at a speed tip ratio of 5 and air velocity of 8 m/s.
3 The erosion profile is V-shaped and the majority of the scars appear near the blade leading edge.
4 The erosion rate increases exponentially with the tip speed ratio for the same mass flow rate and diameter of injected particles.
5 The effect of the mass flow rate of injected particles on erosion rates increases significantly with the tip speed ratio for the same particle diameter.
6 Based on an annual basis, about 5.7 mm maximum erosion depth is expected in some spots of this small-scale wind turbine at 0.003 kg/s and 50 μm of sand flow rate and particle diameter, respectively.

## Data availability

Data will be made available on request.

## CRediT authorship contribution statement

**A.E. Abu El-Maaty:** Writing – original draft, Visualization, Validation, Software, Methodology, Investigation, Formal analysis, Data curation. **H.K. Abdallah:** Writing – original draft, Visualization, Validation, Software, Methodology, Investigation, Formal analysis, Data curation, Conceptualization. **M.A. Kotb:** Writing – original draft, Visualization, Validation, Software, Methodology, Investigation, Formal analysis, Data curation, Conceptualization. **R. Ben-Mansour:** Writing – review & editing, Visualization, Validation, Supervision, Resources, Project administration, Methodology, Investigation, Formal analysis, Data curation, Conceptualization. **E.S. Alatawi:** Writing – review & editing, Writing – original draft, Visualization, Project administration, Methodology, Investigation, Funding acquisition, Conceptualization.

## Declaration of competing interest

The authors declare that they have no known competing financial interests or personal relationships that could have appeared to influence the work reported in this paper.
